# Differences in small intestinal apparent amino acid digestibility of raw bovine, caprine, and ovine milk are explained by gastric amino acid retention in piglets as an infant model

**DOI:** 10.3389/fnut.2023.1226638

**Published:** 2023-09-04

**Authors:** Natalie G. Ahlborn, Carlos A. Montoya, Debashree Roy, Nicole C. Roy, Natascha Stroebinger, Aiqian Ye, Linda M. Samuelsson, Paul J. Moughan, Warren C. McNabb

**Affiliations:** ^1^Riddet Institute, Massey University, Palmerston North, New Zealand; ^2^School of Food and Advanced Technology, Massey University, Palmerston North, New Zealand; ^3^Smart Foods and Bioproducts Group, AgResearch Ltd, Palmerston North, New Zealand; ^4^Department of Human Nutrition, University of Otago, Dunedin, New Zealand; ^5^High-Value Nutrition National Science Challenge, Auckland, New Zealand

**Keywords:** bovine milk, caprine milk, ovine milk, piglet, small intestine, amino acid digestibility, infant

## Abstract

**Background:**

The rate of stomach emptying of milk from different ruminant species differs, suggesting that the small intestinal digestibility of nutrients could also differ across these milk types.

**Objective:**

To determine the small intestinal amino acid (AA) digestibility of raw bovine, caprine, and ovine milk in the piglet as an animal model for the infant.

**Methods:**

Seven-day-old piglets (*n* = 12) consumed either bovine, caprine, or ovine milk diets for 15 days (*n* = 4 piglets/milk). On day 15, fasted piglets received a single meal of fresh raw milk normalized for protein content and containing the indigestible marker titanium dioxide. Entire gastrointestinal tract contents were collected at 210 min postprandially. Apparent AA digestibility (disappearance) in different regions of the small intestine was determined.

**Results:**

On average, 35% of the dietary AAs were apparently taken up in the small intestine during the first 210 min post-feeding, with 67% of the AA digestibility occurring in the first quarter (*p ≤* 0.05) and 33% in the subsequent two quarters. Overall, except for isoleucine, valine, phenylalanine, and tyrosine, the small intestinal apparent digestibility of all AAs at 210 min postprandially in piglets fed ovine milk was, on average, 29% higher (*p ≤* 0.05) than for those fed bovine milk. Except for lysine, there was no difference in the apparent digestibility (*p >* 0.05) of any AAs between piglets fed caprine milk or ovine milk. The apparent digestibility of alanine was higher (*p ≤* 0.05) in piglets fed caprine milk than those fed bovine milk. When apparent digestibility was corrected for gastric AA retention, only small differences in the small intestinal apparent digestibility of AAs were observed across milk types.

**Conclusion:**

Bovine, caprine and ovine milk had different apparent small intestinal AA digestibility at 210 min postprandially. When corrected for gastric AA retention, the differences in apparent digestibility across species largely disappeared. The apparent AA digestibility differed across small intestinal locations.

## 1. Introduction

Globally, bovine milk provides an affordable and accessible source of nutrition. In regions where bovine milk is not readily available, cultural preferences prevail, or self-sufficiency is required, the consumption of non-bovine milk such as ovine, caprine, and camel milk is common (1). In the past decade, Western countries have seen a rise in the consumption of non-bovine milk (2). This trend has been driven by increased demand for specialty foods (1), recognition of the variation in nutrient composition, nutritional benefits (3–5), and a perceived (anecdotal) reduction in digestive discomfort after consumption of non-bovine milk when compared with bovine milk (6, 7). The compositional differences of non-bovine milk have been of interest in developing infant formulas for specialized infant nutrition as an alternative to widely-available bovine milk-based infant formulas (4). Despite this increasing interest in non-bovine milk, there is relatively little knowledge on the digestion and absorption of protein from non-bovine milk, particularly in human infants.

The nutrient composition of bovine, caprine, and ovine milk varies (8). For example, the protein content of ovine milk is higher than that of caprine and bovine milk. The major milk protein fractions, casein and whey, are also in different proportions in bovine, caprine, and ovine milk (9). Thus, the amino acid (AA) profiles of each milk type vary (5, 7).

Previous studies have described the impact of species on the gastric digestion of ruminant milk. For example, the solid phase ‘curd’ formed during *in vitro* gastric digestion of raw ovine and caprine milk has a different protein structure (10), and the curd is also softer (11, 12) than in raw bovine milk. The softer curd formed by the raw caprine and ovine milk results in a faster gastric emptying of protein from the piglet stomach (13). These results for raw milk are consistent with *in vitro* results observed for caprine and bovine milk-based infant formulas (14, 15).

During the gastric digestion of milk, a continuous flow of whey proteins, partially digested casein components (peptides), and AAs is expected to enter the small intestine, where the proteins are further digested, and the AAs are absorbed. Montoya et al. (16) showed that the digestion and absorption of AAs in both the proximal and medial small intestines were positively correlated to the extent of gastric emptying. Similarly, Gaudichon et al. (17) showed that in mini-pigs fed either yoghurt or milk, the kinetics of dietary nitrogen absorption were controlled by the kinetics of dietary nitrogen flow into the small intestine.

Thus, based on the softer curd structure and faster protein emptying rate of ovine milk and caprine milk compared to bovine milk (13), it is hypothesized that there are differences in the amounts of AAs taken up throughout the small intestine in piglets consuming caprine and ovine milk, compared to those consuming bovine milk. However, the digestibility of AAs from raw whole bovine, caprine, and ovine milk from the small intestine has not yet been reported.

This study aimed to determine the apparent digestibility of AAs from bovine, caprine, and ovine milk in different small intestinal regions of piglets at 210 min postprandially. This time point was selected to align with a similar accompanying study where piglets were fed the same milk types, and different gastric digestion and emptying parameters were analyzed (13). Digestibility refers to the disappearance of an AA from the digestive tract and is assumed to equate with the uptake (absorption) of the AA. The piglet is a common animal model for the digestion of milk and infant formula, based on digestive and absorptive similarities from the mouth to the terminal ileum (18–21). Because of their significance for various aspects of human health (22–24), the absorption of physiologically relevant AA groups (essential AA (EAA), branched-chain AA (BCAA), non-essential AA (NEAA), and long neutral AA (LNAA)) was also quantified.

## 2. Materials and methods

### 2.1. Animals, housing, and dietary treatments

**Figure 1:**
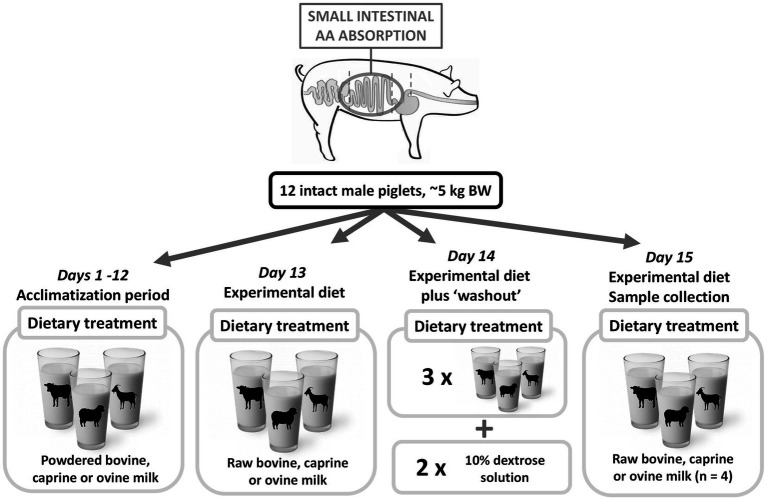
Pictorial representation of the piglet study. Upon arrival, piglets were randomly allocated to a bovine, caprine, or ovine milk group. From days 1–12, piglets were acclimatized to the housing situation, feed frequency, volume, and bottle-feeding method. During this period, piglets consumed reconstituted powdered bovine, caprine, or ovine milk. From day 13, five feeds of the experimental diet (raw bovine, caprine, or ovine milk) were consumed daily. On day 14 (the day prior to sampling), piglets received three meals of the experimental diets, and the final two meals were a 10% dextrose solution as a milk nutrient ‘washout’ period. On day 15, piglets consumed one-morning milk meal and were euthanized at 210 min post-feeding. The contents of the entire gastrointestinal tract were collected in sections. AA, amino acid; BW, bodyweight.

This study was approved by the Massey University Animal Ethics Committee (protocol no. MUAEC 18/97). Locally sourced Large White x Landrace entire male piglets (*n* = 12, 7 days of age, weight 5.17 ± 0.16 kg, mean ± SEM) were housed in individual plastic metabolism crates at the Animal Production Unit of Massey University, Palmerston North. The room was temperature-controlled (28 ± 2°C) and operated under a 16 h:8 h light: dark cycle. There was daily socialization for 1 h, and toys were provided to enrich the experimental conditions for the animals. After arrival, the piglets underwent a 12-day acclimatization period to adapt to bottle-feeding (suckling from a rubber teat), feeding frequency, and feed volume. The piglets received reconstituted spray-dried bovine, caprine, or ovine milk during the adaptation period. On day 13, the piglets received the experimental diets (Figure 1). The experimental diets were fresh raw whole bovine milk (Massey University Dairy Farm No. 4, Palmerston North, New Zealand), fresh raw caprine milk (days 13 and 4: Phoenix Goats, Palmerston North, New Zealand; day 15: Dairy Goat Co-op, Hamilton, New Zealand), and fresh raw ovine milk (Neer Enterprises Ltd., Carterton, New Zealand). Further detail is provided by Roy et al. (13). Raw milk was not provided from the arrival of the animals as sufficient caprine and ovine milk were not available for the entire study.

### 2.2. Experimental design

From days 13–15, the piglets consumed the experimental diets in 5 daily meals at 3.5 h intervals. The volume offered to each animal in each meal was calculated to provide 2 g of protein per kg of body weight (BW) (Table 1). On day 14, piglets received three fresh whole milk meals, followed by two meals consisting of a 10% dextrose solution (Figure 1) to provide an 18 h washout for any residual milk components to leave the gastrointestinal tract prior to the sampling day meal (13). On day 15, fasted piglets (21–22 days old) were bottle-fed one fresh milk meal, which contained 5.1–5.8 mg suspended titanium dioxide (TiO_2_)/g DM and 0.34–0.55 g polyethylene glycol/g DM as indigestible markers to measure the meal flow throughout the gastrointestinal tract (25, 26). The TiO_2_ and the polyethylene glycol were included to allow for measurement of meal flow in the solid and liquid phases, respectively. All piglets consumed their meal in 2 to 3 min and were euthanized 210 min post-feeding. Each piglet was anaesthetized 15 min before its euthanasia time with Zoletil 100 (zolazepam and tiletamine, both 50 mg/mL; Zoetis Inc., Parsippany-Troy Hills, NJ, US) reconstituted with 2.5 mL Ketamine and 2.5 mL Xylazine, both 100 mg/mL (Phoenix Pharm NZ). The final solution contained 50 mg/mL of each drug and was administered at a dose rate of 0.03–0.04 mL of the mixed solution/kg BW by intramuscular injection in the neck. Following sedation, each piglet was intravenously administered a second dose of the cocktail (30 μL/kg BW) to induce deep anesthesia. Once anesthetized, the piglets were euthanized by an intra-cardiac injection of sodium pentobarbitone (0.3 mL of Pentobarb 300/kg BW, Provet NZ Pty Ltd).

**Table 1:** Milk, energy and nutrient allowances and amino acid composition of the fresh raw whole bovine, caprine, or ovine milk provided to piglets in each experimental meal during the last three experimental days^1^^,^^2^.

	Bovine	Caprine	Ovine
	*g/kg bodyweight*		
Fresh milk (g)	55.3^b^	63.1^a^	31.9^c^
Crude protein	2.0 ± 0.02	2.0 ± 0.04	2.0 ± 0.02
Fat–total	2.2 ± 0.08	2.0 ± 0.06	2.0 ± 0.02
Lactose	2.5 ± 0.03^a^	2.5 ± 0.03^a^	1.3 ± 0.01^b^
Dry matter	7.2 ± 0.06^a^	7.1 ± 0.11^a^	5.6 ± 0.02^b^
Gross energy (kcal/kg BW)	41.9 ± 0.59^a^	38.6 ± 0.45^b^	34.3 ± 0.05^c^
	*mg/g protein*		
Isoleucine	30.1 ± 0.61^b^	31.3 ± 0.20^b^	37.0 ± 0.40^a^
Leucine	52.4 ± 1.05^c^	56.5 ± 0.25^b^	74.1 ± 0.95^a^
Valine	34.6 ± 0.73^b^	41.6 ± 0.34^a^	42.9 ± 0.39^a^
*Total BCAA*	*117.0 ± 2.40^c^*	*129.4 ± 0.78^b^*	*154.0 ± 1.73^a^*
Histidine	12.2 ± 0.26^c^	13.2 ± 0.11^b^	18.9 ± 0.25^a^
Lysine	23.8 ± 0.54^b^	25.2 ± 0.14^b^	42.1 ± 0.16^a^
Methionine	12.0 ± 0.17^b^	11.7 ± 0.31^b^	18.2 ± 0.20^a^
Phenylalanine	26.2 ± 0.55^c^	28.7 ± 0.21^b^	34.5 ± 0.55^a^
Threonine	22.0 ± 0.55^c^	27.7 ± 0.29^b^	31.7 ± 0.35^a^
Tryptophan	14.4 ± 0.27	14.8 ± 0.07	15.0 ± 0.16
*Total EAA*	*227.6 ± 4.58^c^*	*250.7 ± 1.37^b^*	*314.4 ± 2.85^a^*
Alanine	17.0 ± 0.40^b^	16.8 ± 0.14^b^	27.7 ± 0.37^a^
Arginine	17.8 ± 0.40^c^	16.5 ± 0.07^b^	25.6 ± 0.17^a^
Asparagine	36.6 ± 1.42^b^	37.1 ± 0.81^b^	58.3 ± 0.63^a^
Cysteine	6.5 ± 0.14	7.5 ± 0.44	7.1 ± 0.33
Glutamine	127.3 ± 3.34^b^	132.7 ± 1.26^b^	172.4 ± 1.93^a^
Serine	27.9 ± 0.71^b^	27.8 ± 0.23^b^	39.4 ± 0.39^a^
Tyrosine	25.5 ± 0.68^c^	23.0 ± 0.12^b^	35.5 ± 0.17^a^
*Total NEAA*	*268.0 ± 7.04^b^*	*270.9 ± 2.56^b^*	*383.7 ± 3.04^a^*
*Total LNAA*	*183.1 ± 3.77^c^*	*195.9 ± 1.04^b^*	*240.9 ± 2.11^a^*

BCAA, branched-chain amino acids; EAA, essential amino acids; NEAA, non-essential amino acids; LNAA, large neutral amino acids.

1 The milk volumes provided to the piglets were calculated to deliver 2 g of protein per kg of bodyweight.

2 Values are means ± SEM, *n* = 3 batches/milk. Means in a row without a common superscript differ (*p* ≤ 0.05) in a comparison between milk types.

Following euthanasia, the abdomen was opened. The esophagus, pylorus, ileal cecal junction, and rectum were clamped. The whole gastrointestinal tract was then dissected. The total gastric contents were collected as previously described by Roy et al. (13). The small intestine was uncoiled, and one clamp was placed approximately 20 cm before the ileal-cecal junction (terminal ileum). The remaining small intestine was separated into two even sections (proximal and distal small intestine; PSI and DSI, respectively). The whole digesta from each section (PSI, DSI, and terminal ileum) were collected using three flushes of distilled water. The large intestine was also uncoiled, and the digesta of the cecum and colon (proximal and distal) were collected as described by Montoya et al. (27). The gastric chyme, small intestine, and large intestinal digesta were immediately frozen on dry ice and stored at −20°C. The samples were then freeze-dried, ground, and sieved (particle size ≤1 mm). All contents were analyzed for TiO_2_ content to determine the transit rate of the meal through the gastrointestinal tract. Insufficient sample volume for each gastrointestinal section prevented the analysis of the polyethylene glycol content.

**Figure 2:**
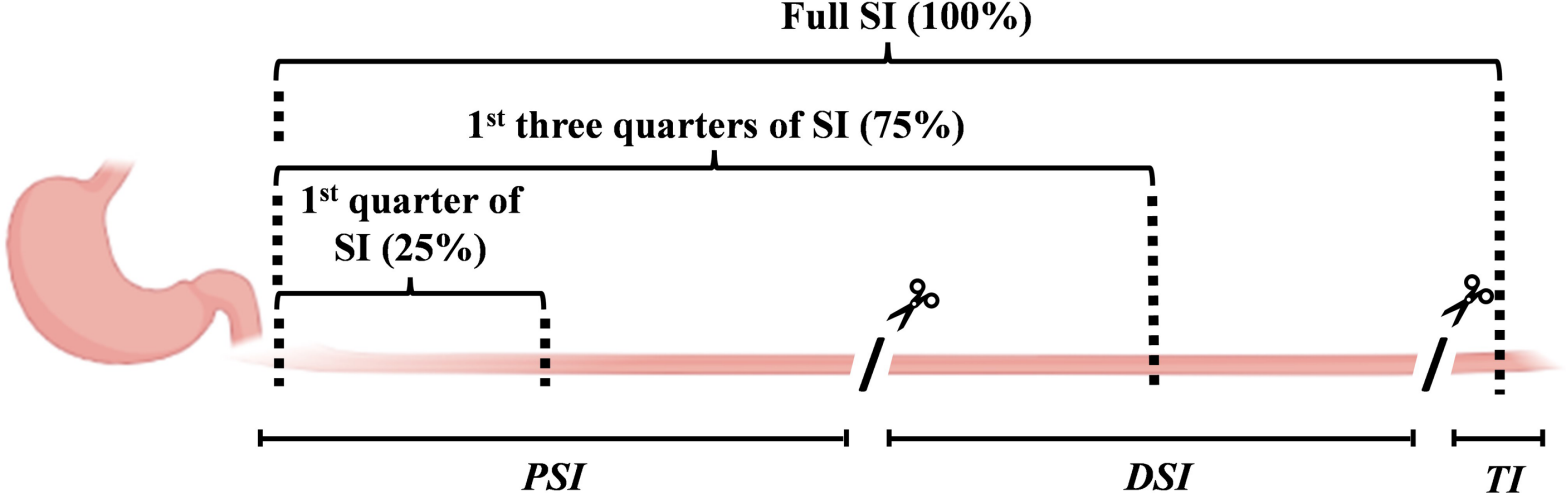
Diagram showing the small intestinal sections. The small intestine was dissected into the proximal and distal small intestine (PSI and DSI) and terminal ileum, and the entire digesta from each section were collected. The amino acid (AA) and TiO_2_ contents of the PSI and DSI digesta were determined, and it was assumed that they represented the values in the middle of each small intestinal location. Thus, the PSI and DSI values are assumed to represent the AA absorption in the first quarter (25%) and the first three-quarters (75%) of the small intestine. The terminal ileal values represent the complete small intestine (100%).

The amounts of AAs remaining in the stomach, PSI, DSI, and terminal ileum were analyzed. The TiO_2_ content of each small intestinal location was then used in conjunction with the AA content to determine the apparent AA digestibility in the PSI, DSI, and terminal ileum. It was assumed that digesta were equally distributed in each location for PSI and DSI. Thus, the PSI values represent the apparent digestibility in the first quarter of the small intestine (i.e., half-way along the PSI, Figure 2) at 210 min post-feeding, while DSI values represent the apparent digestibility of the first three-quarters of the small intestine. The terminal ileal samples represented the apparent digestibility over the entire small intestine.

### 2.3. Chemical analyses

During the study, three batches of fresh raw milk were collected and analyzed for macronutrient and AA contents. Each raw milk type, gastric solid fraction, gastric liquid fraction, PSI, DSI, terminal ileal, cecal, and colonic contents were analyzed for dry matter (DM)[(AOAC 990.19 (28)] and TiO_2_ (29). Each milk type, the gastric solid and liquid fractions, and small intestinal digesta were also analyzed for AA content [24-h HCl hydrolysis, *o*-phthaldialdehyde pre-column derivatization, followed by reverse-phase chromatography (25)]. For the milk types, the cysteine and tryptophan contents were analyzed by performic acid oxidation and alkaline hydrolysis, respectively. The milk types were analyzed in triplicate for the AA content, and the gastrointestinal contents were analyzed in duplicate. Sulfur AAs and tryptophan were not analyzed, as sample volumes were limited. Before the HCl hydrolysis for AA analysis, the gastric samples were defatted using a diethyl ether/petroleum ether extraction as described elsewhere (30) (Supplementary Method S1).

### 2.4. Calculations

On average, 15, 12, and 13% of the TiO_2_ reached the large intestine at 210 min for the bovine, caprine, and ovine milk groups (Supplementary Table S1). However, only three piglets (two fed bovine milk and one fed caprine milk) had enough terminal ileal digesta to analyze both TiO_2_ and AA contents. As smaller amounts of digesta are required for AA analysis compared to the TiO_2_, only the AA analysis was conducted for the other 9 piglets with insufficient terminal ileal sample amounts. The mean TiO_2_ content of the terminal ileal digesta of the two piglets fed bovine milk was used to calculate the amount of AA released into the large intestine for the piglets fed bovine milk with small terminal ileal samples. The same calculation was used for piglets fed caprine and ovine milk, except that for ovine milk, the mean of the three piglets with sufficient terminal ileal digesta was used. Thus, AA digestibility at the terminal ileum was calculated using mean values across species as detailed below, but the results are presented only as indicative values in the Supplementary material.

No correction for endogenous AAs was made; thus, only apparent digestibility values are reported. The amount of TiO_2_ in each gastrointestinal location, the amount of AA present prior to the small intestinal location of interest, the amount of AA in the location of interest, and the amount of AA released post the small intestinal location of interest were considered to calculate the apparent AA digestibility in each small intestinal location. The amount of TiO_2_ that appeared in and was released from each location was calculated as follows (PSI and the first quarter of the small intestine as an example):


$$\begin{array}{l} {\mathrm{TiO}}_{2}\;{\mathrm{content}}_{\mathrm{PSI}}\;\left(g\;\mathrm{on}\;\mathrm{DM}\;\mathrm{basis}\right)=\\ \;\;\;\;\;\;{\mathrm{Total content}}_{\mathrm{PSI}\;\mathrm{digesta}}\;\left(g\;\mathrm{DM}\right)\;\times \;\\ \;\;\;\;\;\;{\mathrm{TiO}}_{2}\;{\mathrm{concentration}}_{\mathrm{PSI}\;\mathrm{digesta}}\;\left(\%\mathrm{DM}\right)/100 \end{array}$$

$${\mathrm{TiO}}_{2}\;{\mathrm{content}}_{\mathrm{until}\;\mathrm{PSI}}={\mathrm{TiO}}_{2}\;{\mathrm{content}}_{\mathrm{Stomach}}+{\mathrm{TiO}}_{2}\;{\mathrm{content}}_{\mathrm{PSI}}$$

$$\begin{array}{l} {\mathrm{TiO}}_{2}\;{\mathrm{content}}_{\mathrm{after}\;\mathrm{PSI}}={\mathrm{TiO}}_{2}\;{\mathrm{content}}_{\mathrm{DSI}}\\ \;\;\;\;\;\;+{\mathrm{TiO}}_{2}\;{\mathrm{content}}_{\mathrm{Terminal ileum}}+{\mathrm{TiO}}_{2}\;{\mathrm{content}}_{\mathrm{Large intestine}} \end{array}$$

The amount of AA present prior (stomach) and in the location of interest (PSI) and released after the location of interest was calculated as follows:


$$\begin{array}{l} \mathrm{AA}\;{\mathrm{content}}_{\mathrm{Stomach}}\;\left(\mathrm{mg}\;\mathrm{on}\;\mathrm{DM}\;\mathrm{basis}\right)=\\ \;\;\;\;\;\;\;\;\;\;\;\;\;\;\;\;\;\mathrm{AA}\;{\mathrm{concentration}}_{\mathrm{Stomach}}\;\left(\%\right)\\ \;\;\;\;\;\;\;\;\;\;\;\;\;\;\;\;\;\times {\mathrm{Total content}}_{\mathrm{Stomach}}\;\left(g\;\mathrm{DM}\right)/100 \end{array}$$

$$\begin{array}{l} \mathrm{AA}\;{\mathrm{content}}_{\mathrm{PSI}}\;\left(\mathrm{mg}\;\mathrm{on}\;\mathrm{DM}\;\mathrm{basis}\right)=\\ \;\;\;\;\;\;\;\;\;\;\;\;\;\;\;\;\;\;\;\mathrm{AA}\;{\mathrm{concentration}}_{\mathrm{PSI}}\;\left(\%\right)\\ \;\;\;\;\;\;\;\;\;\;\;\;\;\;\;\;\;\;\;\times {\mathrm{Total content}}_{\mathrm{PSI}}\;\left(g\;\mathrm{DM}\right)/100 \end{array}$$

$$\begin{array}{l} \mathrm{AA}\;{\mathrm{content}}_{\mathrm{released after 1st quarter small intestine}}\;\left(\mathrm{mg}\right)=\\ \;\;\;\;\;\;\;\left(\mathrm{AA}\;{\mathrm{content}}_{\mathrm{PSI}}\times {\mathrm{TiO}}_{2}\;{\mathrm{content}}_{\mathrm{after}\;\mathrm{PSI}}\right)/\\ \;\;\;\;\;\;\;{\mathrm{TiO}}_{2}\;{\mathrm{content}}_{\mathrm{PSI}\;\mathrm{digesta}} \end{array}$$

These values were used to calculate the AA content recovered in each location, followed by the apparent AA digestibility (or disappearance), as described by Montoya et al. *(unpublished)* (first quarter of the small intestine as an example):


$$\begin{array}{l} \mathrm{AA}\;{\mathrm{unabsorbed}}_{\mathrm{1st quarter small intestine}}\;\left(\mathrm{mg}\right)=\\ \;\;\;\;\;\;\;\;\;\;\;\mathrm{AA}\;{\mathrm{content}}_{\mathrm{Stomach}}+\mathrm{AA}\;{\mathrm{content}}_{\mathrm{PSI}}+\\ \;\;\;\;\;\;\;\;\;\;\;\mathrm{AA}\;{\mathrm{content}}_{\mathrm{released after 1st quarter small intestine}} \end{array}$$

$$\begin{array}{l} \mathrm{Apparent}\;\mathrm{AA}\;\mathrm{digestibility}\;{\left(\%\right)}_{\mathrm{1st quarter}}=\\ \;\;\;\;\;\;\;\;\left(\mathrm{AA}\;{\mathrm{content}}_{\mathrm{diet}}-\mathrm{AA}\;{\mathrm{unabsorbed}}_{\mathrm{1st quarter small intestine}}\right)\\ \;\;\;\;\;\;\;/\mathrm{AA}\;{\mathrm{content}}_{\mathrm{diet}}\times 100 \end{array}$$

The AAs retained in the stomach were subtracted from the dietary AA intake to calculate the apparent digestibility of AAs available for uptake in the small intestine, as AA uptake only occurs in the small intestine. The calculation of unabsorbed AAs described above was adjusted to exclude the AAs retained in the stomach, and the apparent digestibility of AAs available for uptake was calculated as follows:


$$\begin{array}{l} \mathrm{AA}\;{\mathrm{content}}_{\mathrm{entering small intestine}}\;\left(\mathrm{mg}\right)=\\ \;\;\;\;\;\;\;\;\;\;\;\mathrm{AA}\;{\mathrm{content}}_{\mathrm{diet}}-\mathrm{AA}\;{\mathrm{content}}_{\mathrm{stomach}} \end{array}$$

$$\begin{array}{l} \mathrm{AA}\;{\mathrm{unabsorbed}}_{\mathrm{available},\mathrm{1st quarter small intestine}}\;\left(\mathrm{mg}\right)=\\ \;\;\;\;\;\;\;\;\;\;\;\;\;\mathrm{AA}\;{\mathrm{content}}_{\mathrm{PSI}}+\mathrm{AA}\;{\mathrm{content}}_{\mathrm{released after 1st quarter small intestine}} \end{array}$$

$$\begin{array}{l} \mathrm{Apparent}\;\mathrm{AA}\;\mathrm{digestibility}\;{\left(\%\right)}_{\mathrm{available},\mathrm{1st quarter small intestine}}=\\ \;\;\;\;\;\;\;(\mathrm{AA}\;{\mathrm{content}}_{\mathrm{entering small intestine}}-\\ \;\;\;\;\;\;\mathrm{AA}\;{\mathrm{unabsorbed}}_{\mathrm{available},\mathrm{1st quarter small intestine}})\\ \;\;\;\;\;\;/\mathrm{AA}\;{\mathrm{content}}_{\mathrm{entering small intestine}}\times 100 \end{array}$$

The apparent digestibility of physiologically important AA groups (EAA, BCAA, NEAA and LNAA) were calculated using the sum of the AA amounts for each type of AA, followed by the same calculations as the individual AAs.

### 2.5. Statistical analyses

For apparent AA digestibility, statistical analyses were conducted using the PROC MIXED statement of SAS (version 9.4; SAS Institute Inc., Cary, NC, USA; RRID:SCR_008567). A linear mixed model was used to test the effect of species (bovine, caprine, and ovine) and small intestinal location (first quarter, first three-quarters, and whole) and the interaction between species and small intestinal location as fixed effects on individual AA digestibility and EAA EAA, BCAA, NEAA and LNAA digestibility. The pig was used as a random effect. The most appropriate covariance structure (simple, autoregressive, and unstructured) was selected after fitting the model by the restricted maximum likelihood method and comparing the models using the log-likelihood ratio test. Once the covariance structure was selected, the interaction term was removed when it was not significant.

The AA composition of milk and the amount of AA present in the stomach at 210 min were compared across milk types using a one-way ANOVA model (PROC ANOVA procedure of SAS). Batches of milk and piglets were used as experimental units. For the residuals of the model, the normal distribution was evaluated using the ODS Graphics, and the homogeneity of variance was evaluated using the repeated statement by fitting models with the restricted maximum likelihood test and comparing them using the log-likelihood ratio test. When the *F*-value of the analysis of variance was significant (*p* ≤ 0.05), least-square means were compared using an adjusted Tukey test.

## 3. Results

The mean TiO_2_ recovery across all gastrointestinal sections was 98% (Supplementary Table S1). On average, across all milk types, 52% of the consumed TiO_2_ remained in the stomach at 210 min post-feeding. The PSI, DSI, and terminal ileum contained, on average, 9, 24, and 2% of the TiO_2,_ respectively. A further 13% of the consumed TiO_2_ was recovered in the large intestine (cecum, proximal, and distal colon).

Different (*p* ≤ 0.05) amounts of AAs were observed across raw milk types on an mg AA/g protein basis (Table 1). Except for valine, tryptophan, cysteine and proline, ovine milk contained significantly higher (*p ≤* 0.05) amounts of each AA than both bovine and caprine milk. Histidine, leucine, phenylalanine, threonine, arginine, and tyrosine were also present in higher (*p ≤* 0.05) amounts in caprine milk than in bovine milk. There was no difference (*p* > 0.05) in the amount of tryptophan or cysteine between each milk type.

At 210 min post-feeding, a higher (*p ≤* 0.05) ratio (mg retained/mg consumed) of all AAs, except for tyrosine, remained in the stomach of piglets fed bovine milk than for those fed ovine milk (Table 2). For example, 64 and 35% of the dietary leucine consumed remained in the stomach of piglets fed bovine milk or ovine milk, respectively. In particular, lysine appeared to have remained entirely in the stomach of piglets fed bovine milk at 210 min, whereas piglets fed caprine and ovine milk retained 78 and 47% of lysine, respectively. Except for alanine, arginine, and asparagine, there was no difference (*p* > 0.05) in the gastric retention of AA in piglets fed caprine milk compared to piglets fed bovine milk.

**Table 2:** Gastric retention of amino acids in piglets fed bovine, caprine, or ovine milk at 210 min post-feeding^1^.

	Bovine	Caprine	Ovine
*mg retained/mg AA consumed*			
Ile	0.61 ± 0.06^a^	0.42 ± 0.05^ab^	0.37 ± 0.05^b^
Leu	0.64 ± 0.06^a^	0.46 ± 0.05^ab^	0.35 ± 0.05^b^
Val	0.66 ± 0.06^a^	0.47 ± 0.05^ab^	0.41 ± 0.06^b^
*Total BCAA*	*0.64 ± 0.06^a^*	*0.45 ± 0.05^ab^*	*0.37 ± 0.05^b^*
His	0.77 ± 0.07^a^	0.55 ± 0.06^ab^	0.36 ± 0.05^b^
Lys^2^	1.11 ± 0.12^a^	0.78 ± 0.08^ab^	0.47 ± 0.07^b^
Met	0.85 ± 0.08^a^	0.68 ± 0.08^ab^	0.44 ± 0.06^b^
Phe	0.69 ± 0.06^a^	0.49 ± 0.05^ab^	0.39 ± 0.05^b^
Thr	0.64 ± 0.07^a^	0.47 ± 0.05^ab^	0.35 ± 0.05^b^
*Total EAA*	*0.72 ± 0.07^a^*	*0.51 ± 0.06^ab^*	*0.39 ± 0.05^b^*
Ala	0.60 ± 0.06^a^	0.37 ± 0.04^b^	0.30 ± 0.04^b^
Arg	0.77 ± 0.07^a^	0.53 ± 0.05^b^	0.38 ± 0.05^b^
Asp	0.71 ± 0.07^a^	0.48 ± 0.05^b^	0.34 ± 0.05^b^
Glu	0.70 ± 0.07^a^	0.52 ± 0.06^ab^	0.39 ± 0.06^b^
Ser	0.71 ± 0.07^a^	0.50 ± 0.06^ab^	0.37 ± 0.05^b^
Tyr	0.77 ± 0.07	0.51 ± 0.06	0.59 ± 0.08
*Total NEAA*	*0.71 ± 0.07^a^*	*0.49 ± 0.05^ab^*	*0.40 ± 0.05^b^*
*Total LNAA*	*0.67 ± 0.06^a^*	*0.47 ± 0.05^ab^*	*0.42 ± 0.06^b^*

BCAA, branched-chain amino acids; EAA, essential amino acids; NEAA, non-essential amino acids; LNAA, large neutral amino acids.

1 Values are means ± SEM, *n* = 4. Means in a row without a common superscript differ (*p* ≤ 0.05) in a comparison between milk types.

2 The lysine retention (greater than one) for piglets fed bovine milk may be due to residual protein retained in the stomach from the pre-fast milk meal.

On average across species, 67% of the total apparent AA digestibility during the first 210 min post-feeding occurred in the first quarter of the small intestine (Table 3). Excluding lysine, the apparent AA digestibility in the first quarter of the small intestine ranged between 15 and 38%. In contrast, only 10 to 15% of the AA digestibility in the first three-quarters of the small intestine was accounted for by AA digestibility in the second and third quarters of the small intestine. The apparent AA digestibility at the terminal ileum was highly variable and did not differ (*p* > 0.05) from the apparent AA digestibility determined over the proximal small intestinal regions (Supplementary Table S2).

**Table 3:** Overall small intestinal apparent digestibility of amino acids from raw bovine, caprine, and ovine milk, and digestibility in the first quarter (25%) and the first three-quarters (75%) of the small intestine of piglets^1^.

	Milk				Location^2^ (%)			*P* ^3^ ^,^ ^4^	
	Bovine	Caprine	Ovine	SEM	25	75	SEM	Milk	Location
*%*									
Ile	33.2	45.7	49.1	5.2	37.4	47.9	3.2	NS	***
Leu	30.2^b^	40.9^ab^	51.5^a^	5.1	35.5	46.3	3.2	*	**
Val	28.4	40.5	44.4	5.4	32.3	43.2	3.3	NS	***
BCAA	29.5	41.1	46.2	5.3	35.1	42.8	3.3	NS	**
His	16.2^b^	29.8^ab^	49.1^a^	5.8	25.3	38.0	3.7	*	**
Lys^5^	−20.1^b^	1.4^b^	33.4^a^	8.3	−4.0	13.9	5.2	**	**
Met	9.3^b^	14.7^ab^	40.3^a^	6.8	14.7	28.2	4.3	*	**
Phe	24.2	36.9	45.5	5.5	29.6	41.5	3.5	NS	**
Thr	24.5^b^	36.7^ab^	48.1^a^	5.0	30.3	42.6	3.1	*	***
EAA	20.3	33.4	42.8	5.8	27.8	36.5	3.6	NS	**
Ala	28.2^b^	47.3^a^	55.6^a^	3.9	38.3	49.1	2.5	**	***
Arg	9.9^b^	25.6^ab^	41.7^a^	4.8	18.1	33.4	3.2	**	***
Asp	19.5^b^	37.9^ab^	51.6^a^	5.5	30.6	42.1	3.6	**	***
Glu	24.7^b^	36.5^ab^	47.7^a^	5.8	30.9	41.7	3.6	*	**
Ser	20.5^b^	33.8^ab^	46.2^a^	5.5	27.2	39.8	3.5	*	***
Tyr	16.3	33.8	17.4	7.4	15.4	29.5	4.6	NS	**
NEAA	20.6^b^	37.9^ab^	48.0^a^	5.7	28.9	37.3	3.5	*	**
LNAA	19.2	37.4	32.7	6.4	23.6	32.6	4.0	NS	**

SEM, pooled standard error of the mean; BCAA, branched-chain amino acids; EAA, essential amino acids; NEAA, non-essential amino acids; LNAA, large neutral amino acids.

1 Values are means ± SEM, *n* = 4. Means in a row without a common superscript differ (*p* ≤ 0.05) in a comparison between milk types (a, b, c).

2 Apparent digestibility of the terminal ileum is not given as, except for three piglets, the amount of digesta recovered from the terminal ileum was insufficient for both titanium dioxide analysis and amino acid analysis. The terminal ileal absorption values were estimated using averages. The statistical analysis, including the terminal ileum, is shown in Supplementary Table S2.

3 Significance levels are indicated as follows: *p ≤ 0.05; **p < 0.01; ***p < 0.001.

4 There was no significant (*p* > 0.05) milk × location interaction for any of the analyzed amino acids. Thus, the interaction was removed from the final model.

5 The negative lysine digestibility result for piglets fed bovine milk may be due to residual protein retained in the stomach from the pre-fast milk meal (Table 2).

For all individual AAs, both species (milk type) and location had a significant effect on the apparent AA digestibility (*p ≤* 0.05; Table 3). Except for isoleucine, valine, phenylalanine, and tyrosine, the apparent digestibility of other individual AAs from piglets fed ovine milk at 210 min was greater (*p ≤* 0.05) than those fed bovine milk. For example, the apparent digestibility of histidine was 3.1-fold higher in piglets fed ovine milk when compared to those fed bovine milk. A higher apparent digestibility of alanine (+19%) was also observed for piglets fed caprine milk compared to those fed bovine milk (*p <* 0.01). Except for lysine, which had a higher (*p <* 0.01) apparent digestibility in piglets fed ovine milk, there was no difference (*p >* 0.05) in the apparent digestibility of any AAs in piglets fed caprine or ovine milk. The apparent digestibility of NEAAs was higher (*p ≤* 0.05) in piglets fed ovine milk compared to those fed bovine milk.

Except for isoleucine and tyrosine, the apparent amount absorbed (mg AA disappearing per g protein consumed) of all AAs was higher (*p ≤* 0.05) for piglets fed ovine milk than those fed bovine milk at 210 min (Table 4). A higher amount (*p* < 0.01) of valine was apparently absorbed by the piglets fed caprine milk compared to piglets fed bovine milk. For piglets fed ovine milk, a higher amount (*p* < 0.01) of the EAAs histidine, lysine, and methionine, and the NEAAs alanine, arginine, and asparagine was apparently absorbed than for piglets fed caprine milk.

**Table 4:** Overall amount of amino acids from raw bovine, caprine, and ovine milk apparently disappeared, which disappeared in the first quarter (25%) and the first three-quarters (75%) of the small intestine of piglets^1^.

	Milk				Location (%)			*P* ^2^ ^,^ ^3^	
	Bovine	Caprine	Ovine	SEM	25	75	SEM	Milk	Location
*mg AA/g protein consumed*									
Ile	13.8	20.5	22.6	2.3	16.6	21.3	1.4	NS	***
Leu	21.9^b^	33.2^ab^	47.5^a^	4.4	29.7	38.7	2.8	**	**
Val	13.6^b^	24.2^a^	23.7^a^	2.9	17.5	23.5	1.8	*	**
BCAA	47.8^b^	76.3^ab^	88.6^a^	9.8	63.8	78.0	6.1	*	**
His	2.7^b^	5.6^b^	11.5^a^	1.2	5.3	7.9	0.8	**	**
Lys^4^	−6.6^b^	0.5^b^	17.5^a^	3.5	0.2	7.4	2.2	**	***
Met	1.6^b^	2.5^b^	9.1^a^	1.4	3.1	5.6	0.8	**	**
Phe	8.8^b^	15.2^ab^	19.6^a^	2.3	12.1	17.0	1.4	*	**
Thr	7.5^b^	14.6^ab^	19.0^a^	1.9	11.4	16.0	1.2	**	***
EAA	59.9^b^	113.0^ab^	159.4^a^	20.3	95.8	125.7	12.6	*	**
Ala	6.6^b^	11.4^b^	19.2^a^	1.2	10.9	13.9	0.8	***	***
Arg	2.4^b^	6.1^b^	13.3^a^	1.4	5.2	9.3	0.9	***	**
Asp	9.9^b^	20.1^b^	37.4^a^	3.4	19.1	25.9	2.1	***	***
Glu	43.6^b^	69.4^ab^	102.3^a^	11.6	61.1	82.4	7.1	*	**
Ser	7.9^b^	13.5^ab^	22.6^a^	2.5	12.0	17.4	1.5	**	***
Tyr	5.8	11.1	5.2	2.3	5.1	9.6	1.4	NS	**
NEAA	72.2^b^	128.2^ab^	187. 7^a^	23.0	113.4	145.2	14.0	*	**
LNAA	61.6	102.0	110.7	14.6	81.0	101.9	9.0	NS	**

SEM, pooled standard error of the mean; BCAA, branched-chain amino acids; EAA, essential amino acids; NEAA, non-essential amino acids; LNAA, large neutral amino acids.

1 Values are means ± SEM, *n* = 4. Means in a row without a common superscript differ (*p* ≤ 0.05) in a comparison between milk types (a, b, c).

2 Significance levels are indicated as follows: *p ≤ 0.05; **p < 0.01; ***p < 0.001.

3 There was no significant (*p >* 0.05) milk × location interaction for any of the analyzed amino acids. Thus, the interaction was removed from the final model.

4 The negative lysine digestibility result for piglets fed bovine milk may be due to residual protein retained in the stomach from the pre-fast milk meal (Table 2).

As a large portion of the dietary AA content remained in the stomach of all piglets at 210 min, the digestibility of AAs entering the small intestine (i.e., available for uptake) was considered by correcting the dietary AA intake for AAs retained in the stomach and then determining the apparent digestibility of AAs entering the small intestine. On average, across all raw milk types, the apparent digestibility of all AAs entering the small intestine was 81% within the first three-quarters of the small intestine (Table 5). The first quarter of the small intestine was responsible for around 58% of this apparent digestibility. Except for valine and lysine, there was no difference (*p* > 0.05) in the apparent digestibility of AAs entering the small intestine in piglets fed raw bovine, caprine, and ovine milk. Valine entering the small intestine had a higher (*p ≤* 0.05) apparent digestibility in piglets fed caprine milk than those fed ovine milk. The apparent digestibility of lysine entering the small intestine varied (*p ≤* 0.001) across milk types (ovine milk > caprine milk > bovine milk).

**Table 5:** Overall small intestinal apparent digestibility of available amino acids^1^, and digestibility in the first quarter (25%) and first three-quarters (75%) of the small intestine of piglets fed raw bovine, caprine, and ovine milk^2^.

	Milk			Location (%)		*P* ^3^ ^,^ ^4^	
	Bovine	Caprine	Ovine	25	75	Milk	Location
*%*							
Ile	81.6 ± 2.4	83.0 ± 2.4	77.4 ± 2.4	71.1 ± 3.9	90.2 ± 0.8	NS	***
Leu	80.1 ± 2.7	81.4 ± 2.7	77.5 ± 2.7	69.6 ± 4.6	89.8 ± 0.8	NS	***
Val	77.9 ± 2.6^ab^	81.3 ± 2.6^a^	74.0 ± 2.6^b^	67.0 ± 3.6	88.4 ± 0.7	*	***
BCAA	81.5 ± 4.2	75.9 ± 4.2	72.2 ± 4.2	69.3 ± 3.2	83.7 ± 3.2	NS	**
His	65.2 ± 4.0	73.4 ± 4.0	72.1 ± 4.0	55.6 ± 6.3	84.9 ± 1.1	NS	***
Lys	7.0 ± 6.2^c^	34.5 ± 6.2^b^	60.9 ± 6.2^a^	15.9 ± 5.0	52.4 ± 5.0	***	***
Met	47.1 ± 10.6	54.6 ± 10.6	70.1 ± 10.6	40.7 ± 7.0	73.9 ± 7.0	NS	***
Phe	74.1 ± 3.0	78.2 ± 3.0	72.8 ± 3.0	62.9 ± 5.1	87.2 ± 0.9	NS	***
Thr	66.8 ± 2.7	73.3 ± 2.7	70.7 ± 2.7	58.2 ± 4.3	82.3 ± 1.1	NS	***
EAA	65.9 ± 4.1	76.4 ± 4.1	65.2 ± 4.1	65.3 ± 5.0	81.1 ± 1.4	NS	**
Ala	72.1 ± 3.2	75.7 ± 3.2	78.7 ± 3.2	66.0 ± 2.5	85.0 ± 2.5	NS	***
Arg	41.2 ± 6.7	56.1 ± 6.7	66.9 ± 6.7	37.7 ± 4.9	71.7 ± 4.9	NS	***
Asp	66.2 ± 3.0	74.6 ± 3.4	73.5 ± 3.4	58.4 ± 5.4	84.8 ± 1.2	NS	***
Glu	79.1 ± 2.9	81.4 ± 2.9	76.8 ± 2.9	67.7 ± 5.0	90.4 ± 0.9	NS	***
Ser	66.7 ± 3.3	73.0 ± 3.3	69.5 ± 3.3	56.3 ± 5.4	83.2 ± 1.1	NS	***
Tyr	68.2 ± 8.5	69.7 ± 8.5	43.0 ± 8.5	43.5 ± 5.2	77.1 ± 5.2	NS	***
NEAA	70.5 ± 5.0	70.5 ± 5.0	69.7 ± 5.0	61.5 ± 3.8	79.1 ± 3.8	NS	**
LNAA	79.0 ± 4.7	74.2 ± 4.7	68.2 ± 4.7	65.9 ± 3.5	81.6 ± 3.5	NS	**

SEM, pooled standard error of the mean; BCAA, branched-chain amino acids; EAA, essential amino acids; NEAA non-essential amino acids; LNAA, large neutral amino acids.

1 The dietary AA intake was corrected for AAs retained in the stomach as only the AAs entering the small intestine from the stomach are considered available for uptake from the small intestine.

2 Values are means ± SEM, *n* = 4. Means in a row without a common superscript differ (*p ≤* 0.05) in a comparison between milk types (a, b, c).

3 Significance levels are indicated as follows: *p ≤ 0.05; **p < 0.01; ***p < 0.001.

4 There were no significant (*p >* 0.05) milk x location interactions. Thus, the interaction was removed from the final model.

## 4. Discussion

This study is the first to report the apparent digestibility of AAs from raw bovine, caprine, and ovine milk throughout the small intestine. As hypothesized, differences in apparent AA digestibility at 210 min postprandially were observed between piglets fed the different milk types, with generally greater apparent digestibility observed in the first quarter of the small intestine, compared to the subsequent 50% of the small intestine. At 210 min post-feeding, the entire milk meal had not yet transited the whole small intestine, so the current values do not represent the apparent extent of AA digestibility in the small intestine overall but rather a single time point in the kinetics of apparent AA digestibility.

On average, 13% of the TiO_2_ was recovered in the large intestine across all animals, which indicates that part of the meal had transited the small intestine. The estimated apparent AA digestibility in the small intestine was similar to that in the first three-quarters of the small intestine. However, the apparent AA digestibility for the small intestine should be cautiously considered as the amount of digesta collected at the terminal ileum was often too small to accurately measure the AA and TiO_2_ concentrations. Calculations based on mean TiO_2_ values (across species) rather than the actual individual TiO_2_ values could have exaggerated or masked any individual differences in the TiO_2_ content in the terminal ileum, which in turn could have affected the apparent AA digestibility estimates calculated over the small intestine. The small amount of terminal ileal digesta collected could be explained by the highly digestible nature of milk nutrients (31).

In this study, on average, 73, 52, and 39% of the AAs consumed by piglets fed raw bovine, caprine, or ovine milk, respectively, remained in the stomach at 210 min post-feeding, while the gastric TiO_2_ retention was 49, 59 and 47%, respectively. A study using the same animal model and diets as the present study found residual material from the pre-washout milk meal in the stomach of 16 h fasted piglets (13). The piglets fed bovine milk retained 25 and 12% more of the protein consumed than those fed caprine and ovine milk, respectively (Roy et al., unpublished). Thus, the high level of inconsistency between the gastric retentions of the TiO_2_ and the dietary AAs in piglets fed bovine milk could be explained by a larger proportion of the protein from the pre-washout milk meal remaining in the stomach at 210 min.

In the same piglet study discussed previously, Roy et al. (13) showed that the gastric emptying rate of dietary protein was faster for piglets fed raw caprine and ovine milk than bovine milk (7.1 and 8.2 vs. 3.6% dietary protein/min x 10^−3^, respectively). The gastric emptying of protein was associated with the structure (protein and lipids) and the strength of the gastric curd formed by each milk type. A similar association was observed between curd structure and curd strength with the gastric protein emptying rate in a study of growing pigs fed processed bovine milk (30). Thus, the greater proportion of AAs remaining in the stomach of piglets fed bovine milk likely remained entrapped in the denser and firmer gastric curd.

As expected, the AA gastric retention at 210 min (mean across milk types) was inversely correlated (*r* = −0.97) to apparent AA digestibility in the first three-quarters of the small intestine. Thus, the greater gastric emptying of caprine and ovine milk proteins compared to bovine milk protein partially explains their higher apparent AA digestibility at 210 min. It has been demonstrated in pigs fed beef muscle protein that the amount of digested nitrogen entering the small intestine correlates positively to the apparent AA digestion and absorption in the first half of the small intestine (16).

To better understand the disappearance of milk proteins, the AAs retained in the stomach were subtracted from those consumed to calculate the apparent digestibility of AAs that entered the small intestine (AAs available for uptake; Table 5). Based on correlations reported elsewhere (16), the apparent digestibility of AAs entering the small intestine was used as a proxy for the degree of protein hydrolysis to estimate whether the degree of hydrolysis of the milk proteins entering the small intestine differed across ruminant milk types. Proteins with a greater degree of gastric hydrolysis are expected to have greater apparent AA digestibility. The small differences across milk types (species) and the lack of significant interactions between raw milk type and small intestinal location for the apparent digestibility of AAs available for uptake suggest that the protein of the different milk types entered the small intestine with a similar degree of hydrolysis after transiting the stomach.

Taken together, the differences in gastric AA retention and similarities in apparent digestibility of available AAs suggest that, when the same amount of protein was consumed (2 g/kg BW), the differences observed in apparent AA digestibility across piglets fed the different milk types are mainly ascribed to the amounts of AA retained in the stomach. It is important to note that the current results apply to infants and cannot be extrapolated to adult humans as the infant gastrointestinal tract is still comparatively immature (32, 33). Further, the current results cannot be extrapolated to consuming the same volume of each ruminant milk type, as the protein content across milk types differs. Further research is warranted to determine the rate of apparent AA digestibility across milk types and in adult humans.

The results from the present study, together with those reported by Roy et al. (13), show that the structural changes of milk in the stomach result in differences in the rate of release of protein into the small intestine, which in turns affects the apparent digestibility of AAs. These findings corroborate the suggestions raised in preclinical and clinical human studies, where it is proposed that gastric emptying and small intestinal uptake are related to differences in the appearance of blood plasma AA over time across milk types and dairy products (34–37). It is important to note that in terms of the rate of plasma AA appearance, other mechanisms (e.g., splanchnic metabolism) can also influence the relationship between AA uptake and appearance in the peripheral circulation (38, 39), so more research is required to understand this relationship in the context of milk from different ruminant species.

The amounts of AAs which apparently disappeared at 210 min were, in general, higher for piglets fed raw ovine milk than for those fed bovine milk. Based on other studies, it could be expected that the differences in the amounts of AAs which apparently disappeared at 210 min have implications for various aspects of protein metabolism in infants. The present results point to differences across the ruminant milk types in the kinetics of AA digestibility from milk, and it remains to be established whether such differences translate to differences in overall small intestinal AA disappearance.

For example, other aspects of protein metabolism observed in adult humans, such as postprandial protein deposition (36), are also expected to be modulated by the amount of AA absorbed in infants. In addition, the hippocampal gene expression of some receptors of the neurotransmitter gamma-aminobutyric acid was higher in piglets fed raw ovine milk compared to those fed raw bovine milk (40), which has implications for infant brain development and early cognitive function (41, 42).

It should be pointed out that in reporting apparent AA digestibility, the endogenous losses for each protein were assumed to be similar, and attempts to calculate the true AA digestibility of the milk types by using reported endogenous losses in the stomach and small intestine were limited by a lack of appropriate literature. It is recommended that the true digestibility of AAs from bovine, caprine and ovine milk is investigated, as differences in small intestinal endogenous AA flows may influence the apparent digestibility results presented here.

The presently reported results were collected using raw milk; however, as milk is usually processed to improve safety and preservation, raw milk is not commonly consumed. Thus, further research is warranted to determine the effect of processing on various parameters such as gastric emptying and AA digestibility. Information on small intestinal AA digestibility patterns across milk types and processing methods is expected to provide evidence to develop dairy products with benefits for specific aspects of human health.

## 5. Conclusion

The present study found that at 210 min post-feeding, on average, 22, 38, and 46% of the AAs consumed apparently disappeared within the first three-quarters of the small intestine of piglets fed bovine, caprine, and ovine milk, respectively. For most AAs, at least two-thirds of the apparent AA disappearance occurred in the first quarter of the small intestine. In general, the apparent small intestinal digestibility of AAs at 210 min was higher for piglets fed ovine milk than bovine milk. The difference in the apparent AA digestibility was related to the amount of AA remaining in the stomach. When comparing the apparent digestibility of the AA entering the small intestine, there were no differences in apparent AA digestibility across species for most AAs.

This study provides a new understanding of the AA digestibility of raw bovine, caprine, and ovine milk in the small intestine of infants. However, considering that milk is commonly processed before consumption or in preparation for dairy product production, further research is needed to understand the effect of processing on AA digestibility throughout the small intestine.

## Data availability statement

The raw data supporting the conclusions of this article will be made available by the authors, without undue reservation.

## Ethics statement

The animal study was approved by Massey University Animal Ethics Committee. The study was conducted in accordance with the local legislation and institutional requirements.

## Author contributions

CM and PM designed the study, which was resourced by funding acquired by WM, PM, and NR. DR, CM, and NS conducted the animal study. NA analyzed the data and performed statistical analysis. NA wrote the first draft of the manuscript, which was critically reviewed and edited by CM, PM, NR, and WM. CM, NR, WM, AY, and LS provided the PhD supervision. All authors have read and approved the final manuscript.

## Funding

This study was supported by New Zealand Tertiary Education Commission (TEC) Centre of Research Excellence (CoRE) funding and the Ministry of Business, Innovation and Employment through the New Zealand Milks Mean More (NZ3M) Endeavor program. NZ3M is a collaborative partnership between the Riddet Institute, AgResearch Ltd., the University of Auckland and the University of Otago, hosted and led by the Riddet Institute at Massey University. NA was supported by a Ph.D fellowship from the Riddet Institute through the NZ3M program.

## Conflict of interest

CM, and LS were employed by AgResearch Ltd.

The remaining authors declare that the research was conducted in the absence of any commercial or financial relationships that could be construed as a potential conflict of interest.

## Publisher’s note

All claims expressed in this article are solely those of the authors and do not necessarily represent those of their affiliated organizations, or those of the publisher, the editors and the reviewers. Any product that may be evaluated in this article, or claim that may be made by its manufacturer, is not guaranteed or endorsed by the publisher.
