# Supplementary material for: Differences in small intestinal apparent amino acid digestibility of raw bovine, caprine, and ovine milk are explained by gastric amino acid retention in piglets as an infant model
Source: Front Nutr. 2023 Sep 4;10:1226638. doi: 10.3389/fnut.2023.1226638 (PMC10507170; doi:10.3389/fnut.2023.1226638)
Supplement: Supplementary file 1 [file Table_1.docx]

***Table S1.*** TiO_2_ content recovered in the gastrointestinal tract of piglets fed raw whole bovine, caprine, or ovine milk at 210 min postprandially^1^.

|  | Bovine | Caprine | Ovine |
| --- | --- | --- | --- |
|  | *mg (% consumed)* | | |
| Consumed | 113.8 ± 13.4  (100%) | 114.3 ± 14.8  (100%) | 122.7 ± 10.5  (100%) |
| Stomach | 54.9 ± 4.8  (49%) | 67.0 ± 8.3  (59%) | 59.3 ± 12.7  (47%) |
| Proximal small intestine | 11.0 ± 1.0  (10%) | 8.0 ± 0.8  (7%) | 11.4 ± 1.2  (10%) |
| Distal small intestine | 28.1 ± 4.7  (24%) | 24.1 ± 6.9  (21%) | 33.1 ± 6.3  (28%) |
| Terminal ileum^2^ | 2.3 ± 0.6  (2%) | 1.3 ± 0.5  (1%) | 2.9 ± 0.8  (2%) |
| Cecum | 1.7 ± 0.3  (2%) | 1.5 ± 0.8  (1%) | 3.4 ± 1.0  (3%) |
| Proximal colon | 11.5 ± 4.1  (9%) | 10.0 ± 2.5  (9%) | 11.6 ± 3.2  (10%) |
| Distal colon | 4.1 ± 2.4  (3%) | 2.3 ± 1.1  (2%) | 1.1 ± 0.3  (1%) |
| Recovered | 113.5  (99.7%) | 114.2  (99.9%) | 122.8  (100%) |

^1^ Values are means ± SEM, *n* = 4 piglets. There was no differences (*P* > 0.05) in the amount of TiO_2_ recovered from piglets fed each milk type in any location.

^2^ The TiO_2_ recovered from the terminal ileum of piglets fed each ruminant milk was estimated using the TiO_2_ recovered from the terminal ileal digesta of two piglets fed cow milk, and one fed goat milk.
