# Supplementary material for: Differences in small intestinal apparent amino acid digestibility of raw bovine, caprine, and ovine milk are explained by gastric amino acid retention in piglets as an infant model
Source: Front Nutr. 2023 Sep 4;10:1226638. doi: 10.3389/fnut.2023.1226638 (PMC10507170; doi:10.3389/fnut.2023.1226638)
Supplement: Supplementary file 2 [file Table_2.DOCX]

***Table S2.*** Overall small intestinal apparent digestibility (%) of dietary amino acids from raw

bovine, caprine, and ovine milk, and digestibility in the first quarter (25%), first three-quarters (75%), and for the whole small intestine (including terminal ileal material (100%))^1,2^.

|  | Milk | | |  |  | Location (%) | | |  |  | *P*^4,5^ | | |
| --- | --- | --- | --- | --- | --- | --- | --- | --- | --- | --- | --- | --- | --- |
|  | Bovine | Caprine | Ovine | SEM |  | 25 | 75 | 100^3^ | SEM |  | Milk | Location |  |
| *%* | | | | | | | | | | | | |  |
| Ile | 33.8 | 46.9 | 49.7 | 5.2 |  | 37.4^y^ | 47.9^x^ | 45.2^xy^ | 3.2 |  | NS | *** |  |
| Leu | 29.9^b^ | 43.2^ab^ | 52.4^a^ | 5.2 |  | 35.5^y^ | 46.3^x^ | 43.6^xy^ | 3.2 |  | * | *** |  |
| Val | 28.9 | 41.8 | 44.9 | 5.4 |  | 32.3^y^ | 43.2^x^ | 40.2^xy^ | 3.4 |  | NS | *** |  |
| BCAA | 29.4 | 43.6 | 48.0 | 5.3 |  | 35.1 ^y^ | 42.8 ^x^ | 43.0 ^x^ | 3.2 |  | NS | * |  |
| His | 17.3^b^ | 31.4^ab^ | 50.1^a^ | 5.9 |  | 25.3^y^ | 38.0^x^ | 35.3^xy^ | 3.7 |  | * | *** |  |
| Lys^6^ | -18.6^c^ | 3.6^ab^ | 35.0^a^ | 8.4 |  | -4.0^y^ | 13.9^x^ | 10.1^xy^ | 4.5 |  | ** | *** |  |
| Met | 10.4^b^ | 16.3^ab^ | 41.3^a^ | 6.9 |  | 14.7^y^ | 28.2^x^ | 25.2^xy^ | 4.4 |  | * | *** |  |
| Phe | 24.9 | 38.4 | 46.3 | 5.6 |  | 29.6^y^ | 41.5^x^ | 38.5^xy^ | 3.5 |  | NS | *** |  |
| Thr | 24.2^b^ | 38.1^ab^ | 48.6^a^ | 5.0 |  | 30.3^y^ | 42.6^x^ | 38.0^xy^ | 3.2 |  | * | *** |  |
| EAA | 20.2 | 36.2 | 44.7 | 5.8 |  | 27.8 ^y^ | 36.5 ^x^ | 36.8 ^x^ | 3.5 |  | * | * |  |
| Ala | 27.7^b^ | 48.2^a^ | 56.0^a^ | 4.0 |  | 38.3^y^ | 49.1^x^ | 44.6^xy^ | 2.5 |  | ** | *** |  |
| Arg | 10.0^b^ | 27.3^ab^ | 42.5^a^ | 4.9 |  | 18.1^y^ | 33.4^x^ | 28.4^x^ | 3.2 |  | ** | *** |  |
| Asp | 19.4^b^ | 39.2^ab^ | 52.3^a^ | 5.5 |  | 30.6^y^ | 42.1^x^ | 38.3^xy^ | 3.3 |  | ** | *** |  |
| Glu | 25.6^b^ | 37.9^ab^ | 48.6^a^ | 5.8 |  | 30.9^y^ | 42.0^x^ | 39.5^xy^ | 3.6 |  | * | *** |  |
| Ser | 20.9^b^ | 35.2^ab^ | 46.9^a^ | 5.5 |  | 27.2^y^ | 39.8^x^ | 35.9^xy^ | 3.5 |  | * | *** |  |
| Tyr | 17.4 | 35.5 | 18.0 | 7.4 |  | 15.4^y^ | 29.5^x^ | 25.9^xy^ | 4.6 |  | NS | *** |  |
| NEAA | 20.6 | 37.9 | 45.3 | 5.7 |  | 28.9 ^y^ | 37.3 ^x^ | 37.5 ^x^ | 3.4 |  | * | ** |  |
| LNAA | 19.2 | 37.4 | 32.7 | 6.4 |  | 23.6 ^y^ | 32.6 ^x^ | 33.1 ^x^ | 3.9 |  | NS | * |  |

SEM, standard error of the mean; EAA, essential amino acids; BCAA, branched-chain amino acids; LNAA, long neutral amino acids.

^1^ Values are means ± SEM, n = 4. Means in a row without a common superscript differ in a comparison between milk types (a, b, c) or location (x, y, z).

^2^ Except for three piglets, there were insufficient quantities of terminal ileum digesta for both marker and amino acid analysis. Therefore, the terminal ileal absorption values were estimated using averages.

^3^ Based on the similarities in AA digestibility at three-quarters of the small intestine and the small intestine overall, no further absorption was predicted for any amino acid after the first ¾ of the small intestine

^4^ Significance levels are indicated as follows: * = *P* ≤ 0.05; ** = *P* *<* 0.01; *** = *P* *<* 0.001.

^5^ There were no significant (*P >* 0.05) milk x location interactions. Thus, the interaction was removed from the final model.
^6^ The negative lysine digestibility result for piglets fed cow milk may be due to residual protein retained in the stomach from the pre-fast milk meal (Table 2).
