# Supplementary material for: Differences in small intestinal apparent amino acid digestibility of raw bovine, caprine, and ovine milk are explained by gastric amino acid retention in piglets as an infant model
Source: Front Nutr. 2023 Sep 4;10:1226638. doi: 10.3389/fnut.2023.1226638 (PMC10507170; doi:10.3389/fnut.2023.1226638)
Supplement: Supplementary file 3 [file Data_Sheet_1.docx]

***Method S1.*** Gastric chyme defatting

Freeze-dried and ground gastric curd (solid fraction) and liquid fraction were weighed (1 g) in a glass tube before addition of petroleum ether and diethyl ether at a 1:5:5 ratio. Solvents were added, vortexed for 30 s, stood for 5 min, and centrifuged at 3,000 rpm for 7 min at room temperature. The solvent fraction was removed using a water-vacuum system. The same procedure was repeated twice to remove residual solvent. The samples were then incubated at 105°C for one hour, cooled, and the remaining lipid-free sample material was weighed.
